# Csf2 Attenuated Sepsis-Induced Acute Kidney Injury by Promoting Alternative Macrophage Transition

**DOI:** 10.3389/fimmu.2020.01415

**Published:** 2020-07-07

**Authors:** Yiming Li, Pan Zhai, Yawen Zheng, Jing Zhang, John A. Kellum, Zhiyong Peng

**Affiliations:** ^1^Department of Critical Care Medicine, Zhongnan Hospital of Wuhan University, Wuhan, China; ^2^Department of Neurology, Hubei Province Hospital of Tradition Chinese Medicine, Wuhan, China; ^3^Department of Urological Organ Transplantation, The Second Xiangya Hospital of Central South University, Changsha, China; ^4^Center of Critical Care Nephrology, Department of Critical Care Medicine, University of Pittsburgh School of Medicine, Pittsburgh, PA, United States

**Keywords:** sepsis, acute kidney injury, macrophage transition, Csf2, cytokine-based therapy

## Abstract

Sepsis is a systemic inflammatory state that occurs in response to infection and significantly increases mortality in combination with acute kidney injury (AKI). Macrophages accumulate in the kidney after injury and undergo a transition from a proinflammatory (M1) phenotype to an alternatively activated (M2) phenotype that is required for normal repair. However, the specific signals that regulate the transition from the M1 to M2 phenotype *in vivo* are unknown. Here, we found an unexpected role of Colony stimulating factor 2 (Csf2) in controlling macrophage transition *in vitro* and in a mouse model of sepsis induced by cecal ligation and puncture (CLP). We first co-cultured human M1 macrophages with HK-2 cells and characterized cytokine/chemokine profiles via Luminex. Of the cytokines and chemokines that were overexpressed in medium from M1 macrophages cocultured with human kidney-2 (HK-2) cells compared with that from M1 macrophages cultured alone, Csf2 and IL6 showed the greatest increases. Csf2 was exclusively secreted by HK-2 cells but not by M1 macrophages. Furthermore, recombinant human Csf2 protein promoted transition of M1 macrophages to the M2 phenotype in a dose and time-dependent manner. The apoptosis and reactive oxygen species (ROS) release induced by M1 macrophages in HK-2 cells was attenuated after exposure to exogenous Csf2. In addition, the switch from the proinflammatory M1 phenotype to the M2 phenotype occurred via the p-Stat5 pathway, which was activated by Csf2. Importantly, we found that intraperitoneal injection of a Csf2-neutralizing antibody after CLP aggravated kidney injury and suppressed tubular proliferation, subsequently decreasing survival. However, administration of recombinant mouse Csf2 protein could rescue mice with sepsis. Together, our results indicate that Csf2 plays critical roles in regulating macrophage transition via activation of p-STAT5. These data form a foundation upon which new therapeutic strategies can be designed to improve the therapeutic efficacy of cytokine-based treatments for sepsis-induced AKI.

## Introduction

Sepsis is a complex clinical syndrome characterized by a systemic inflammatory response to infection. Acute kidney injury (AKI) is one of the most frequent and serious complications and contributes to high mortality. Up to 60% of cases with sepsis are complicated with AKI, and approximately half of AKI cases are related to sepsis (1–3). The disease burden from AKI results in an estimated $10 billion of additional costs for the health care system in the United States (4), and severe AKI is associated with a mortality of 45–70% (2, 5–7).

Pathogen-associated molecular patterns (PAMPs) activate resident macrophages and kidney parenchymal cells, leading to the secretion of proinflammatory cytokines and chemokines that cause nonspecific tissue damage (8). An excessive inflammatory response and oxidative stress are considered to be the main mechanisms of septic AKI (9). Kidney monocytes have been implicated in pathogenesis and healing in mouse models of AKI; these cells infiltrate the injured kidney shortly after neutrophils in the early stage of injury, differentiate into macrophages, and contribute to tissue injury (10). Various experimental models suggest that extended inflammatory macrophage activation augments inflammation. Increased inflammatory cell infiltration and cytokine/chemokine release contribute to AKI (11), and many of these proinflammatory mediators can trigger cell death pathways. Furthermore, the removal of these activated cells and cytokines results in improved survival (12).

Indeed, prolonged inflammatory activity results in further tissue damage, ultimately inhibiting the reparative phase of injury resolution (13). The inflammatory milieu is reversed by the actions of reparative cells via secretion of anti-inflammatory cytokines and pro-proliferative signals. In disease states and injury, macrophages expand and play distinct but important roles in the immune response. They acquire a spectrum of phenotypes that range from highly inflammatory at the beginning of disease to highly reparative toward the resolution of injury. M1 macrophages exacerbate injury by producing proinflammatory cytokines (14), resulting in the recruitment of other inflammatory cells (15). M1 macrophages also secrete antipathogenic molecules, such as NO generated by inducible nitric oxide synthase (iNOS) and ROS, both of which can also induce mitochondrial damage and apoptosis (16). Activated M2 macrophages recognize and downregulate high levels of inflammatory proteins. M2 macrophages also promote the deposition of extracellular matrix by producing arginase, and they inhibit inflammatory immune cell activity via the secretion of resolvents, lipoxins, matrix metalloproteinases, and TGFβ, which target and cleave chemokines and chemoattractants (17). Moreover, depletion of macrophages in the reparative stages results in a significant increase in kidney injury biomarkers (18). However, these two disparate roles of macrophages, from inflammation and injury in the early stage to tissue repair and remodeling in the recovery stage, remain to be further studied. Therefore, identification of the trigger that promotes the transformation from the M1 phenotype to the M2 phenotype may provide therapeutic targets to relieve inflammation and promote tissue repair.

In this study, we showed that macrophage polarization from M1 to M2 in the kidney promotes repair and attenuates septic AKI. M1 macrophages were cocultured with human kidney cells to assess the interaction between these two cell types. Csf2 secreted by kidney tubular cells promoted the repair of tubular cell injury by inducing M0/M1-to-M2 transformation *in vitro*. To further determine how tubular cells promote M2 transformation, we examined the JAK-p-STAT5 pathway in cultured macrophages. Finally, a neutralizing Csf2 antibody and recombinant Csf2 protein were injected intraperitoneally to evaluate alternative macrophage activation and tubular cell injury during the repair stage after AKI. These findings not only address our knowledge gaps regarding the detrimental roles of M1 in AKI but also identify an unexpected role of Csf2 in regulating M1-to-M2 transformation. Modulating Csf2 signaling could improve the therapeutic efficacy of cytokine-based therapies in septic AKI.

## Materials and Methods

### Cell Culture

**Figure 1:**
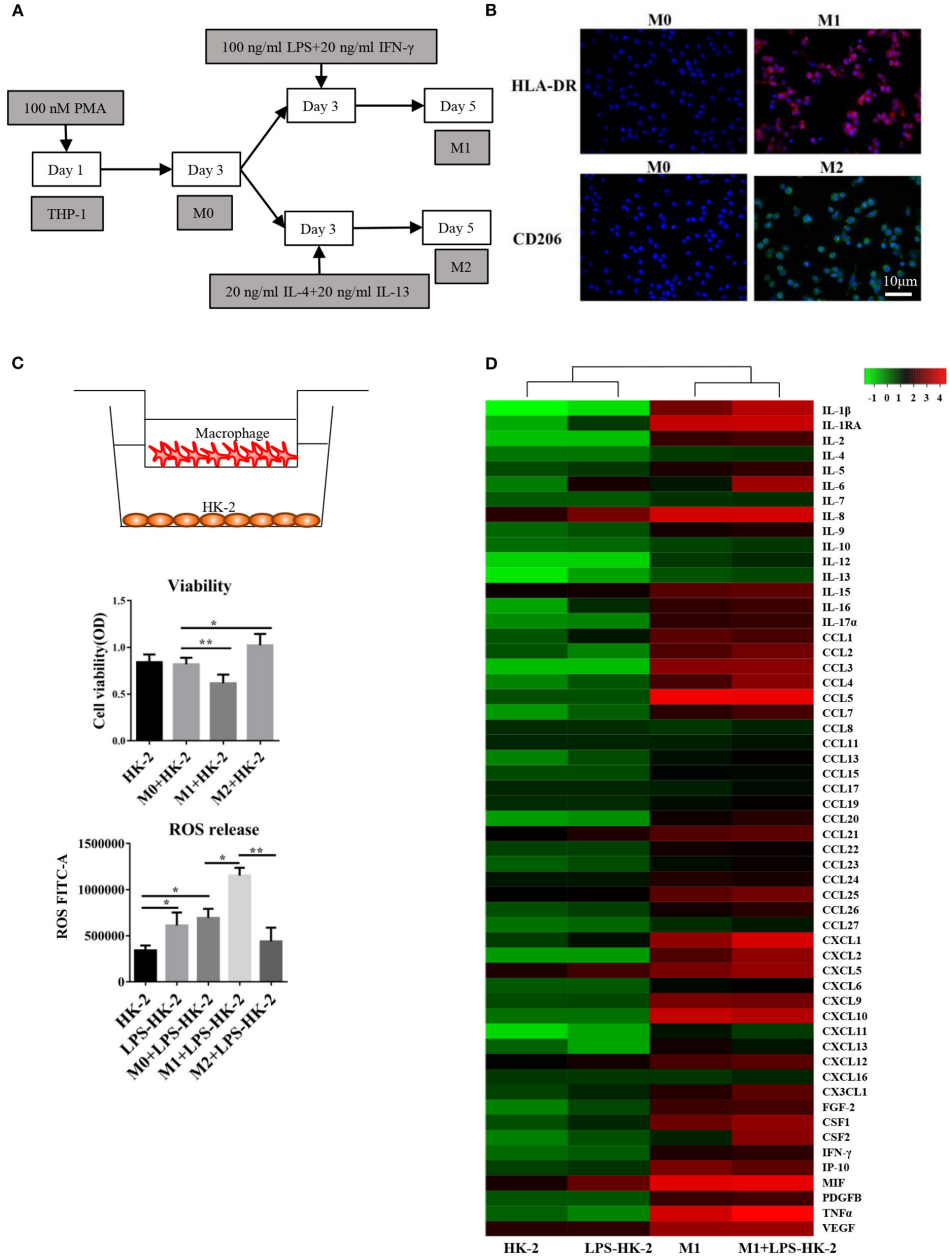
Differentiation and cytokine profiles of M1 macrophages. **(A)** Schematic diagram showing the *in vitro* differentiation of THP-1 cells into M1 and M2 macrophages. **(B)** Images show HLA-DR (red, M1 marker) and CD206 (green, M2 marker) expression after differentiation. DAPI was used to counterstain nuclei. Slides were directly visualized using an Olympus fluorescence microscope at a 20X magnification, scale bar = 20 μm. Representative images were from two independent experiments. **(C)** M0/M1/M2 macrophages were plated on 0.4 mm Transwell inserts and grown over a 2-day period. HK-2/LPS-HK2 cells were plated in 24-wells. The HK-2 cells were subjected to a viability assay. ROS release was detected in HK-2 cells by flow cytometry. LPS-HK2 cells were pretreated with LPS. Data are represented as the mean ± SD from *n* = 3 experiments. The significance of differences was tested using Student's *t*-test. ^*^p < 0.05 and ^**^p < 0.01. **(D)** Cytokines and chemokines in the supernatant after HK-2/LPS-HK-2/M1 culture or M1+ LPS-HK-2 coculture were assessed with a 27-plex cytokine panel and a 40-plex chemokine panel. Twelve cytokines overlapped between these two detection kits. Fifty-five cytokines and chemokines are shown in the heatmap. The Z-score was used to depict the variation between different samples. The culture media were pooled from three experiments and then subjected to Luminex.

The human kidney-2 (HK-2) and THP-1 cell lines were purchased from the Cell Bank of the Chinese Academy of Sciences. We followed the cell culture methods of Li et al. (19). Then, 10 μg/mL LPS (Sigma, L3129) was used to treat HK-2 cells for 18 h. Next, 100 nM phorbol-12-myristate-13-acetate (PMA) was applied to induce THP-1 cell differentiation into M0 macrophages. As shown in Figure 1A, different methods were used to promote M1 or M2 differentiation. For the coculture experiments, the LPS-treated HK-2 cells were washed with PBS and then cocultured with M1 or M0 macrophages in 5% fetal bovine serum (FBS)-containing medium for several additional days. The Transwell chambers (0.4 μm pore size) used for coculture were purchased from Thermo Fisher. HLA-DR is a marker of M1 macrophages. CD206, CD163 and IL-10 are M2 markers. CD68 is usually used to label M0 macrophages.

### Cytokine and Chemokine Assays

Multiplex kits for measuring cytokines and chemokines were purchased from Bio-Rad (Austin, TX, USA). Plates were measured and analyzed with Bio-Plex Manager version 6.1 (Luminex, Austin, TX, USA), sold by Wayen Biotechnologies, Inc. (Shanghai, China). The 27-plex cytokine panel and 40-plex chemokine panel kits were used to measure the concentrations of cytokines and chemokines in the supernatant. Twelve cytokines overlapped between these two detection kits.

### Cell Viability Assays

M1 macrophages were treated with recombinant human Csf2 (215-GM-050, Novus, USA). Cell viability was then tested with a Cell Counting Kit 8 (Dojindo Molecular Technologies, Japan) at 48 h after coculture. The absorbance at 450 nm was measured using a Thermo Scientific Microplate Reader.

### Flow Cytometry Assay

At 48 h after coculture with M1 macrophages for 48 h, HK-2 cells were collected. The cells were fixed with 70% ethanol. The fixed cells were washed with PBS and then treated with DCFH-DA according to the Reactive Oxygen Species Assay Kit (Yeasen, Shanghai, China). In the apoptosis experiment, Annexin V-FITC/PI (Annexin V-FITC/PI Apoptosis Detection Kit, Yeasen, Shanghai, China) was used to label apoptotic cells. The stained cells were analyzed using a Becton Dickinson flow cytometer (Franklin Lakes, NJ).

### Cell Migration Assays

Transwell chambers (8-μm pore size, Corning, USA) were used to perform the cell migration assays. M1 macrophages (2 × 10^5^) were seeded in the insert of the chamber. After coculture with LPS-treated HK-2 cells for 48 h, cells that migrated to the lower surface were stained with crystal violet (Sigma-Aldrich, St. Louis, MO) and photographed.

### ELISA

TIMP2 and Csf2 levels in the cell culture supernatant were measured with an ELISA kit (Bio-Swamp Life Science, Shanghai, China) according to the manufacturer's instructions. To detect IL-10 and TNF-α in kidney tissues, the kidney cortex was homogenized, and the protein concentration was determined using the Coomassie blue method. IL-10 and TNF-α levels in tissue and cell culture supernatant were measured with ELISA kits. Each sample was measured in triplicate.

### RNA Extraction and qRT-PCR

Total RNA was extracted from HK-2 cells, macrophages and kidney tissues using the YPH EASY spin tissue/cell RNA quick extraction kit (YPH, Beijing, China). Furthermore, cDNA was synthesized with the ReverTra Ace Kit (Toyobo, Osaka, Japan). The SYBR Green real-time polymerase chain reaction (PCR) kit was used to detect the expression of target mRNA. The human primer sequences were as follows: Csf2 sense, 5′-TACCTTTGTTGCAGCTGCTG−3′, and anti-sense, 5′-CACCACCCACTGTTCGCTG−3′; CD163 sense, 5′-TTTGTCAACTTGAGTCCCTTCAC−3′, and anti-sense, 5′-TCCCGCTACACTTGTTTTCAC−3′; and IL10 sense, 5′-GACTTTAAGGGTTACCTGGGTTG−3′, and anti-sense, 5′-TCACATGCGCCTTGATGTCTG-3′. The mouse primer sequences were as follows: Csf2 sense, 5′-GGCCTTGGAAGCATGTAGAGG-3′, and anti-sense, 5′-GGAGAACTCGTTAGAGACGACTT−3′; CD163 sense, 5′-ATGGGTGGACACAGAATGGTT-3', and anti-sense, 5′-CAGGAGCGTTAGTGACAGCAG-3'; and IL10 sense, 5′-GCTCTTACTGACTGGCATGAG-3′, and anti-sense, 5′-CGCAGCTCTAGGAGCATGTG-3′.

### Western Blot Analysis

The cells or kidney tissues were lysed, and lysates were prepared. Membranes were incubated with primary Abs specific for the following proteins: Csf2 (Abcam, ab54429), CD206 (Proteintech, 18704-1-AP), ARG1 (Proteintech, 16001-1-AP), t-Stat5 (Abcam, ab227687), p-STAT5 (Cell Signaling Technology, mAb #4322), and Jak2 (Proteintech, 17670-1-AP). Anti-glyceraldehyde 3-phosphate dehydrogenase (GAPDH, Proteintech, 60004-1-Ig) was used as a loading control.

### Immunofluorescence

Immunofluorescence staining of the kidney was performed using paraffin or frozen sections as previously described (19). The sections were incubated with HLA-DR and CD206 antibodies (1:100). The slides were then exposed to a Cy3-conjugated secondary antibody for HLA-DR (red) and a fluorescein isothiocyanate (FITC)-conjugated secondary antibody for CD206 (green). The sections were visualized using fluorescence microscopy (Olympus, Tokyo, Japan).

### Sepsis-Induced CLP Model

C57BL/6 mice (male, 10–12 weeks old) were subjected to CLP to induce sepsis as previously described (20). Briefly, the animals were anesthetized with isoflurane. Under aseptic conditions, a 2-cm midline laparotomy was created below the diaphragm to expose the caecum. The cecum was ligated at the middle with a 5-0 silk suture and punctured twice with a 22-gauge needle. The caecum was then squeezed gently to extrude a small amount of feces through the perforation site. Animals were resuscitated with 1 ml of saline subcutaneously (s.c.) after CLP. We followed the methods of Li et al. (19). To minimize variability across experiments, the CLP procedure was always performed by the same investigator. All experiments were performed in accordance with the Animal Care and Use Committee of Wuhan University.

### Csf2 Antibody and Csf2 Protein Administration to Mice

For Csf2 neutralization experiments, five doses of 300 mg of anti-mouse Csf2-neutralizing antibody (BioXcell, BE0259-5MG) (13) or rat IgG isotype control (BioXcell, BE0089-5MG) in 100 μl of PBS were injected intraperitoneally daily starting 24 h after CLP. For rescue experiments, recombinant mouse Csf2 protein (2.5 μg/mouse/d, NOVUS) (21) in 100 μl of PBS was intraperitoneally administered five times after CLP. We used the 7-day survival rate as the endpoint for our survival study.

### Evaluation of Renal Function

Mice were sacrificed at 48 h following Csf2 antibody or Csf2 protein administration. Heparinized blood was centrifuged to separate the plasma. Serum creatinine (Scr) and blood urea nitrogen (BUN) were detected using commercial kit reagents (Institute of Jiancheng Bioengineering, Nanjing, China).

### H&E Staining

Kidneys were fixed in a 10% buffered formaldehyde solution. The tissue was processed routinely and paraffin embedded. Paraffin blocks were cut using a microtome at a thickness of 4 μm, and the sections were deparaffinized, hydrated, and stained with hematoxylin and eosin (H&E). Photomicrographs were obtained randomly from the kidney renal cortex.

### TUNEL Assay and Immunohistochemistry

Forty-eight hours following Csf2 antibody or Csf2 protein administration, kidney tissues were harvested and fixed with 4% paraformaldehyde for 30 min at room temperature. The TUNEL assay was performed using a TUNEL Apoptosis Detection Kit (Yeason, Shanghai, China) according to the manufacturer's instructions. Briefly, 4-μm kidney sections were deparaffinized and rehydrated. Then, the sections were incubated with TUNEL reagent mixture for 30 min at room temperature and washed with PBS three times for 5 min each time. 4′,6-Diamidino-2-phenylindole was used to stain nuclei. For immunohistochemistry, a Ki67 antibody (Proteintech, 27309-1-AP) was used to stain the tissue sections. The numbers of total cells and Ki67+ cells were determined.

### Statistical Analysis

All tests were analyzed with SPSS version 20.0 or GraphPad Prism 8.0. Student's *t*-test was used for comparisons between the two groups. Survival data were analyzed using the log-rank test. In all comparisons, *P* < 0.05 indicated statistical significance. The data are presented as the mean and standard deviation.

## Results

### Changes in Cytokines/Chemokines Produced by Macrophages and HK-2 in Response to LPS

An *in vitro* model based on differentiation of the THP-1 human monocyte cell line into M1 or M2 macrophages was first established. THP-1 cells grew in suspension with a rounded morphology, and they became attached to the plate in the presence of PMA. The schematic diagram shows that THP-1 cells differentiated into M1 and M2 macrophages (Figure 1A). Immunofluorescence staining for the macrophage markers HLA-DR (M1 marker) and CD206 (M2 marker) was used to confirm the phenotype of these macrophages. After LPS and IFN-γ treatment, the macrophages expressed high levels of HLA-DR (M1, red), while exposure to IL4 and IL13 resulted in the expression of CD206 (M2) (Figure 1B). Following activation, M0, M1 or M2 macrophages were cocultured with HK-2 cells pretreated with LPS for 18 h. Cell viability of HK-2 cells was significantly higher after coculture with M2 macrophages and lower after coculture with M1 macrophages. Similarly, the release of ROS was increased in the M1+ LPS-HK2 group compared to the M0+ LPS-HK2 group (Figure 1C).

Macrophages at various tissue sites have different cytokine profiles (22). Thus, we first measured a panel of 55 cytokines and chemokines in conditioned medium (Figure 1D). The absolute value of cytokines and chemokines were shown in Supplementary Table 1. Most proinflammatory cytokines, such as IL8 and MIF, were increased in HK-2 cells after exposure to LPS. Moreover, CXCL1, IL-1β and tumor necrosis factor (TNF) α were secreted at higher levels by M1 macrophages cocultured with HK-2 cells than by M1 macrophages cultured alone. Of the cytokines and chemokines that were overexpressed in medium from M1 + LPS-HK-2 cultures compared with medium from M1 macrophages cultured alone, IL-6 and Csf2 showed the greatest increases (147.5- and 114.3-fold, respectively, for M1 + LPS-HK-2 vs. M1). The changes in cytokine profiles between M1 macrophages cultured alone and macrophages cocultured with LPS-HK-2 cells confirmed that crosstalk existed between macrophages and HK-2 cells. These cytokine and chemokine array results also indicated that several cytokines might regulate the differentiation of macrophages. Csf2 was reported to facilitate the development of the immune system and promote defense against infections (23). These results indicate that macrophages interacted with kidney cells and that Csf2 was increased in the coculture medium.

### Csf2 Was Derived Exclusively From HK-2 Cells and Decreased HK-2 Cell Apoptosis

**Figure 2:**
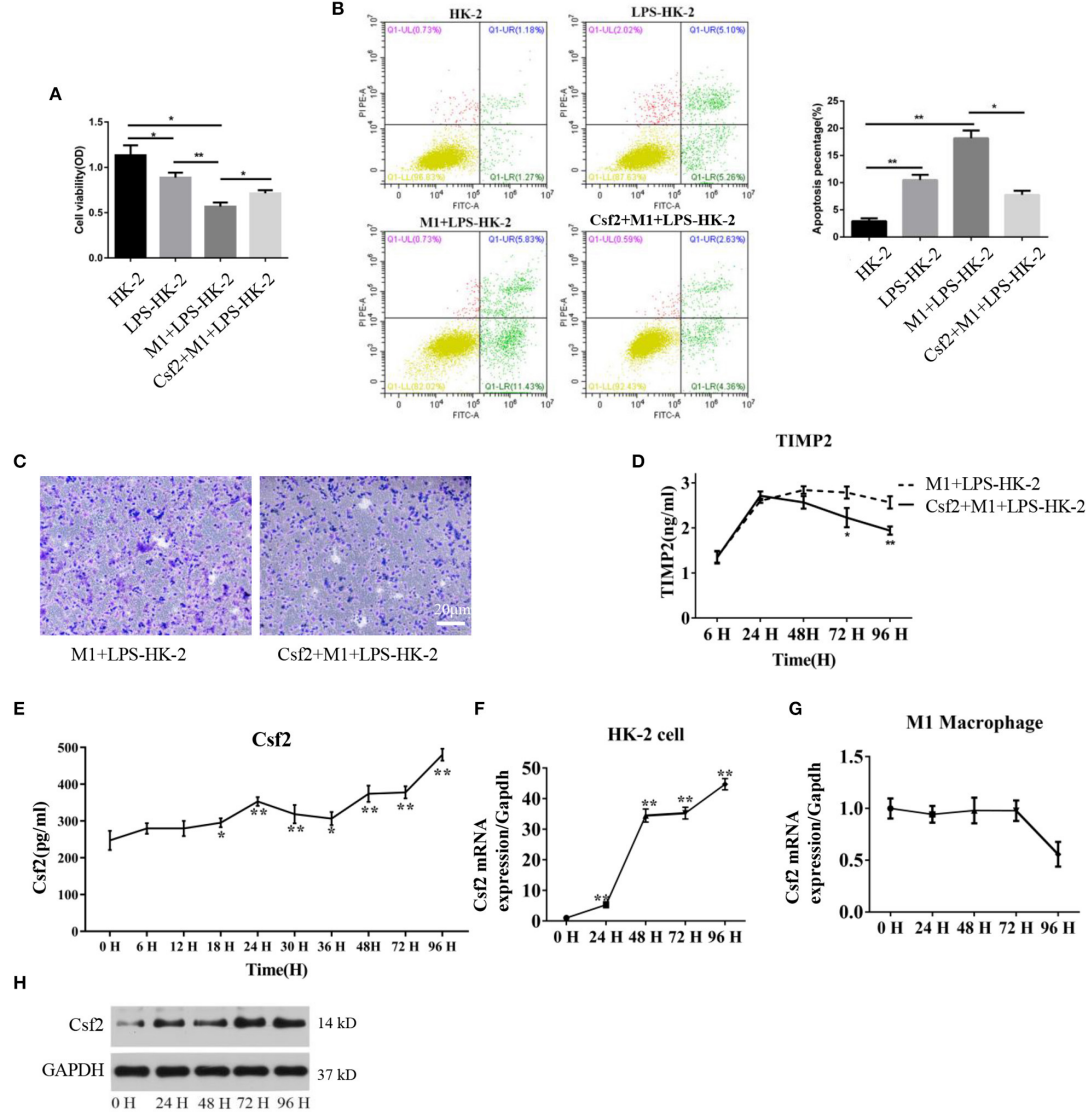
Csf2 secreted from HK-2 cells decreases HK-2 cell apoptosis. **(A)** HK-2 cells were treated with LPS, followed by washing with PBS (LPS-HK-2). LPS-HK-2 cells were cocultured with M1 (M1 + LPS-HK-2 group) or with 25 ng/mL Csf2 (Csf2 + M1 + LPS-HK-2 group) for 18 h, followed by the cell viability test. Three independent experiments were performed. **(B)** Apoptosis of HK-2 cells treated as described above was analyzed by flow cytometry. The apoptosis percentage (Annexin V-FITC+) was shown in the right panel. Three independent experiments were performed. *p < 0.05 and **p < 0.01; Student's *t*-test. **(C)** M1 macrophages were seeded in the upper chamber. After coculture with LPS-treated HK-2 cells for 48 h, M1 macrophages that migrated to the lower chamber were stained with crystal violet. Representative photos were obtained from three independent experiments at a 200 × magnification. **(D)** The concentration of TIMP2 in M1and LPS-HK-2 culture media with or without Csf2 was detected by ELISA. **(E)** The concentration of Csf2 in the medium of LPS-stimulated HK-2 cells cocultured with M1 macrophages was detected by ELISA. Three independent experiments were performed. *p < 0.05 and **p < 0.01; Student's *t*-test. **(F,G)** The mRNA of Csf2 was measured independently in HK-2 cells and M1 macrophages after coculture for the indicated times. Data are the mean ± SD of 3 separate experiments. The significance of differences was tested using Student's *t*-test. *p < 0.05. **(H)** Western blot analysis was used to detect the content of Csf2 in HK-2 cells after coculture with M1 macrophages. Three independent experiments were performed.

Csf2 is considered a mediator by which T cells communicate with myeloid populations during tissue inflammation (24). To verify the specific role of Csf2 in sepsis, exogenous Csf2 was added to the medium in the M1 macrophage and HK-2 cell coculture system. The viability of HK-2 cells was decreased after exposure to M1 macrophages. However, additional Csf2 treatment rescued the viability of HK-2 cells (Figure 2A). The apoptosis rate was assayed to determine the antiapoptotic effect of Csf2. The total apoptosis percentage, including early apoptosis (Annexin V-positive and PI-negative) and late apoptosis (Annexin V and PI double-positive), of HK-2 cells was increased in the M1 + LPS-HK-2 group (10.49% vs. 18.18%, LPS-HK-2 vs. M1 + LPS-HK-2), but the apoptosis percentage decreased to 7.76% after exposure to additional Csf2 (Figure 2B). Next, the Transwell assay was performed to investigate the ability of Csf2 to attract M1 macrophages. Fewer M1 macrophages migrated toward the HK-2 cells when Csf2 was added to the medium (Figure 2C). Moreover, the concentration of TIMP-2, a biomarker of kidney cell stress, was also reduced (Figure 2D). We next measured the concentration of Csf2 in this coculture system. We found that Csf2 secretion increased significantly in a time-dependent manner (Figure 2E). This increased Csf2 could be derived from either HK-2 cells, M1 macrophages, or both. To determine the source of Csf2, qRT-PCR was performed to detect the relative expression of Csf2 in HK-2 cells and M1 macrophages. Interestingly, Csf2 was mainly secreted by HK-2 cells but not M1 macrophages (Figures 2F,G). This increase in Csf2 expression was confirmed by Western blotting in HK-2 cells after coculture with M1 macrophages (Figure 2H). Taken together, these findings clearly demonstrated that Csf2 could decrease HK-2 cell apoptosis and improve cell viability and that Csf2 was derived exclusively from HK-2 cells.

### Csf2 Mediated Macrophage Transition Toward an M2 Phenotype via the p-STAT5 Pathway

**Figure 3:**
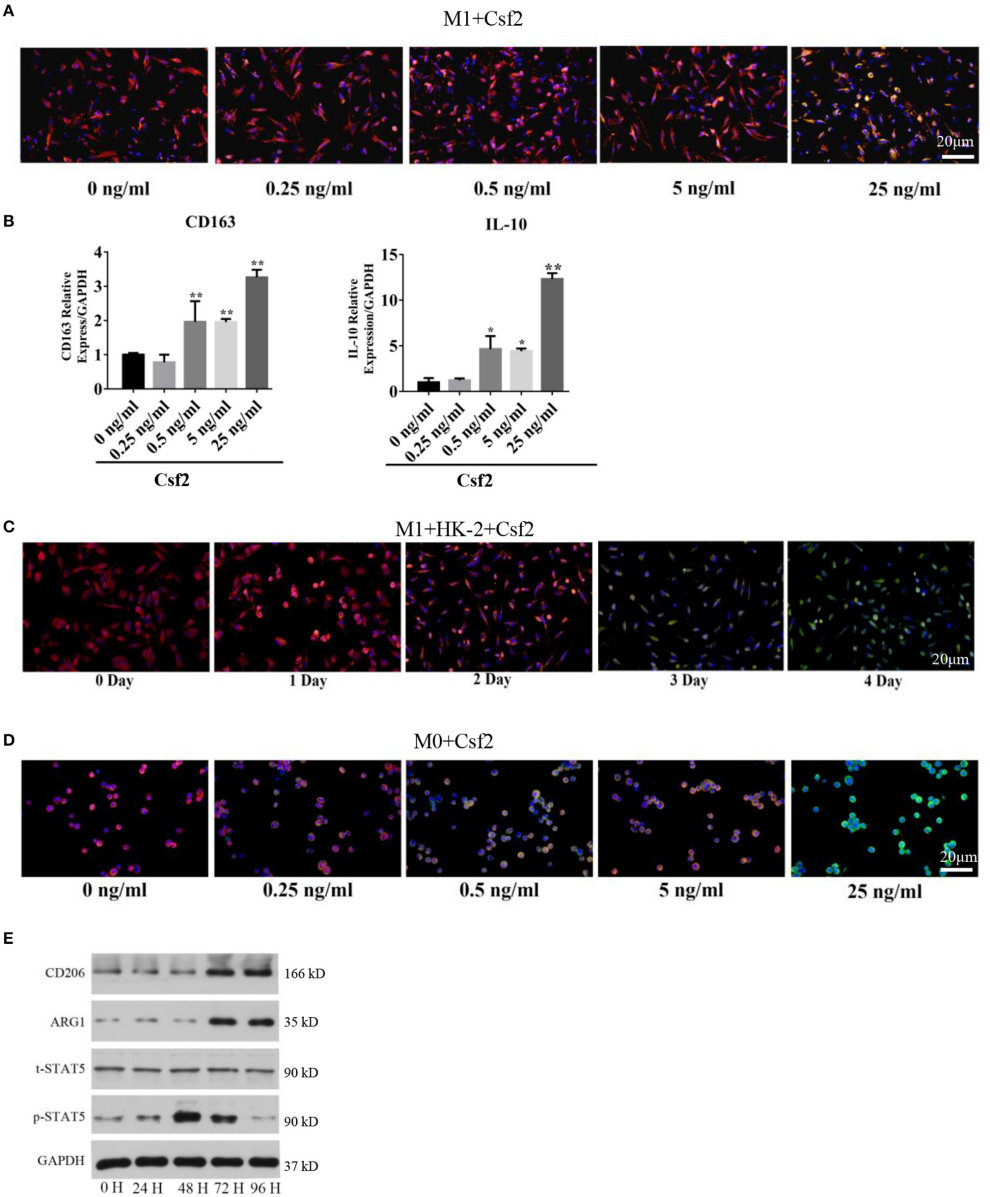
Csf2 mediated alternative activation via the p-STAT5 pathway. **(A)** Representative immunofluorescence images of M1 macrophages showing the expression of HLA-DR (red, M1 marker) and CD206 (green, M2 marker) with Csf2 stimulation at different doses for 48 h. Slides were directly visualized using an Olympus fluorescence microscope at a 20X magnification, scale bar = 20 μm. **(B)** qRT-PCR was used to detect the mRNA levels of CD163 and IL-10 in M1 macrophages exposed to different doses of Csf2. Data are the mean ± SD of three separate experiments. The significance of differences was tested using Student's *t*-test. *p < 0.05. **(C)** Representative immunofluorescence images of M1 macrophages showing the expression of HLA-DR (red, M1 marker) and CD206 (green, M2 marker) after coculture with LPS-HK-2 for 4 days. Slides were directly visualized using an Olympus fluorescence microscope at a 20X magnification, scale bar = 20 μm. **(D)** Representative immunofluorescence images of M0 macrophages showing the expression of CD68 (red, M0 marker) and CD206 (green, M2 marker) following Csf2 stimulation at different doses for 48 h. Slides were directly visualized using an Olympus fluorescence microscope at a 20X magnification, scale bar = 20 μm. **(E)** Western blot analysis was used to detect CD206 (M2 marker), ARG1 (M2 marker), total STAT5 and phosphorylated STAT5 in M1 macrophages at the indicated times. Three independent experiments were performed.

To study the effect of Csf2 on macrophages, M1 macrophages were treated with Csf2 for 72 h. We detected changes in the expression of HLA-DR (red, M1 macrophage) and CD206 (green, M2 macrophage). CD206 fluorescence was increased at the cell membrane of M1 macrophages (Figure 3A). Among the different doses of Csf2, the mRNA expression of the M2 phenotype markers CD163 and IL-10 was increased, with the highest increase at a dose of 25 ng/ml (Figure 3B). Additionally, immunofluorescence staining showed that the expression of CD206 on the M1 macrophage surface increased over time following coculture with HK-2 cells after Csf2 exposure (Figure 3C). We then wondered whether Csf2 had a similar effect on M0 macrophages. The number of CD68 (red, M0 marker) and CD206 (green, M2 macrophage) double-positive cells increased in a dose-dependent manner in response to Csf2 (from 0.5 to 25 ng/ml, Figure 3D). This finding indicates that M1 macrophages differentiated into M2 macrophages in the presence of Csf2.

Macrophage reprogramming is mediated by the JNK, PI3K/Akt, Notch, JAK/STAT, TGF-β, and TLR/NF-κB pathways (25). The JAK-STAT pathway plays an important role in regulating macrophage transformation (26, 27). To determine whether the JAK-STAT signaling pathway was involved in tubular cell-mediated macrophage activation, we analyzed STAT5 signaling pathways in M1 macrophages at multiple time points after Csf2 treatment. Phosphorylated STAT5 levels were much higher in macrophages after 48-72 h of Csf2 exposure (Figure 3E). Moreover, the M2 markers CD206 and ARG1 were also increased. These findings support the hypothesis that M1 macrophages shift toward an M2 phenotype through the STAT5 pathway when exposed to mediators such as Csf2.

### Effect of Csf2 on Kidney Function and Survival Rates in a Sepsis Model

**Figure 4:**
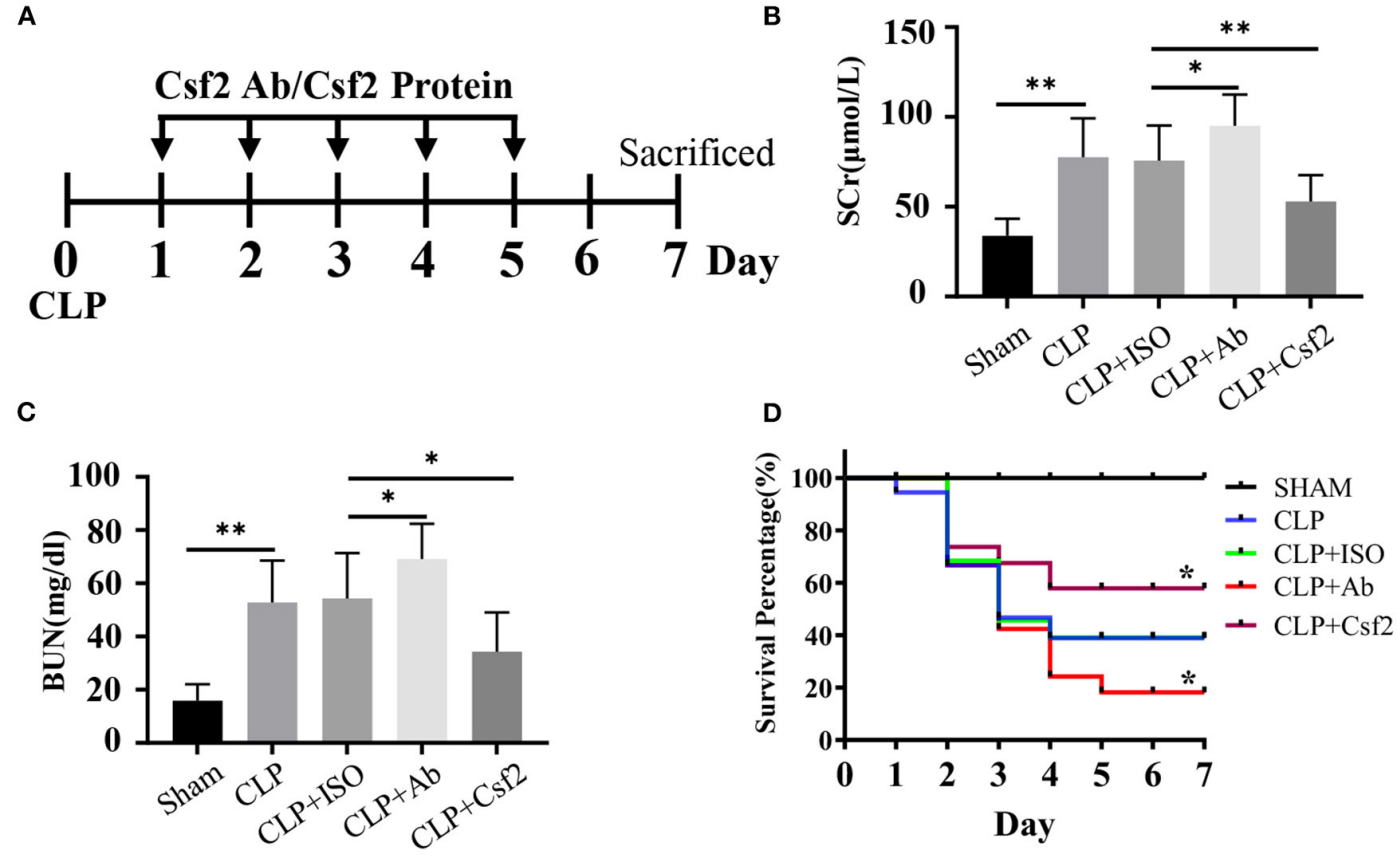
Effect of Csf2 on kidney function and survival rates in a sepsis model. **(A)** Schematic timeline of the experiment. Mice were subjected to CLP and then injected with a neutralizing Csf2 antibody, recombinant Csf2 protein or an isotype antibody daily starting 24 h after CLP. Five doses of 300 mg/mouse/d anti-mouse Csf2-neutralizing antibody or 2.5 μg/mouse/d recombinant mouse Csf2 protein were injected intraperitoneally. **(B,C)** Mice were sacrificed at 48 h following Csf2 antibody or Csf2 protein administration. Scr and BUN levels were determined in the SHAM and CLP groups. Data are shown as the mean ± SD of 10 mice and were pooled from three independent experiments, *p < 0.05; ** *p* < 0.01, two-tailed unpaired Student's t-test. **(D)** Seven-day survival was observed, n=20/group, *p < 0.05; CLP+ Ab vs. CLP + ISO; CLP + Csf2 vs. CLP + ISO. The significance of differences was tested using the log-rank test.

A previous study showed that CLP minimally affected survival in Csf2^−/−^ mice while markedly reducing survival in wild-type mice (28). We hypothesized that the Csf2 antibody may aggravate AKI and decrease survival in a sepsis model. To test our hypothesis, a Csf2-neutralizing antibody or recombinant mouse Csf2 protein was intraperitoneally injected five times starting at 24 h after CLP. As shown in Figure 4A, five doses of anti-mouse Csf2-neutralizing antibody or recombinant mouse Csf2 protein were injected intraperitoneally. Serum was harvested 48 h following Csf2 antibody or Csf2 protein administration. Scr and BUN levels in the CLP group were increased compared with those in the SHAM group. Notably, intraperitoneal injection of the Csf2 antibody significantly exacerbated renal dysfunction, as evaluated by Scr and BUN levels. However, Scr and BUN levels were decreased after Csf2 injection compared with IgG isotype injection (Figures 4B,C). The survival rate decreased to 15.8% at 7 days after Csf2 antibody injection, compared with 36.8% after Csf2 isotype injection. Surprisingly, injection of Csf2 after CLP significantly reduced mortality (Figure 4D). These data revealed that blockade of Csf2 after CLP aggravated kidney injury and decreased survival and that Csf2 could rescue sepsis in mice.

### Blockade of Csf2 After CLP Inhibited Kidney Macrophage Transition and Reduced Tubular Cell Proliferation

**Figure 5:**
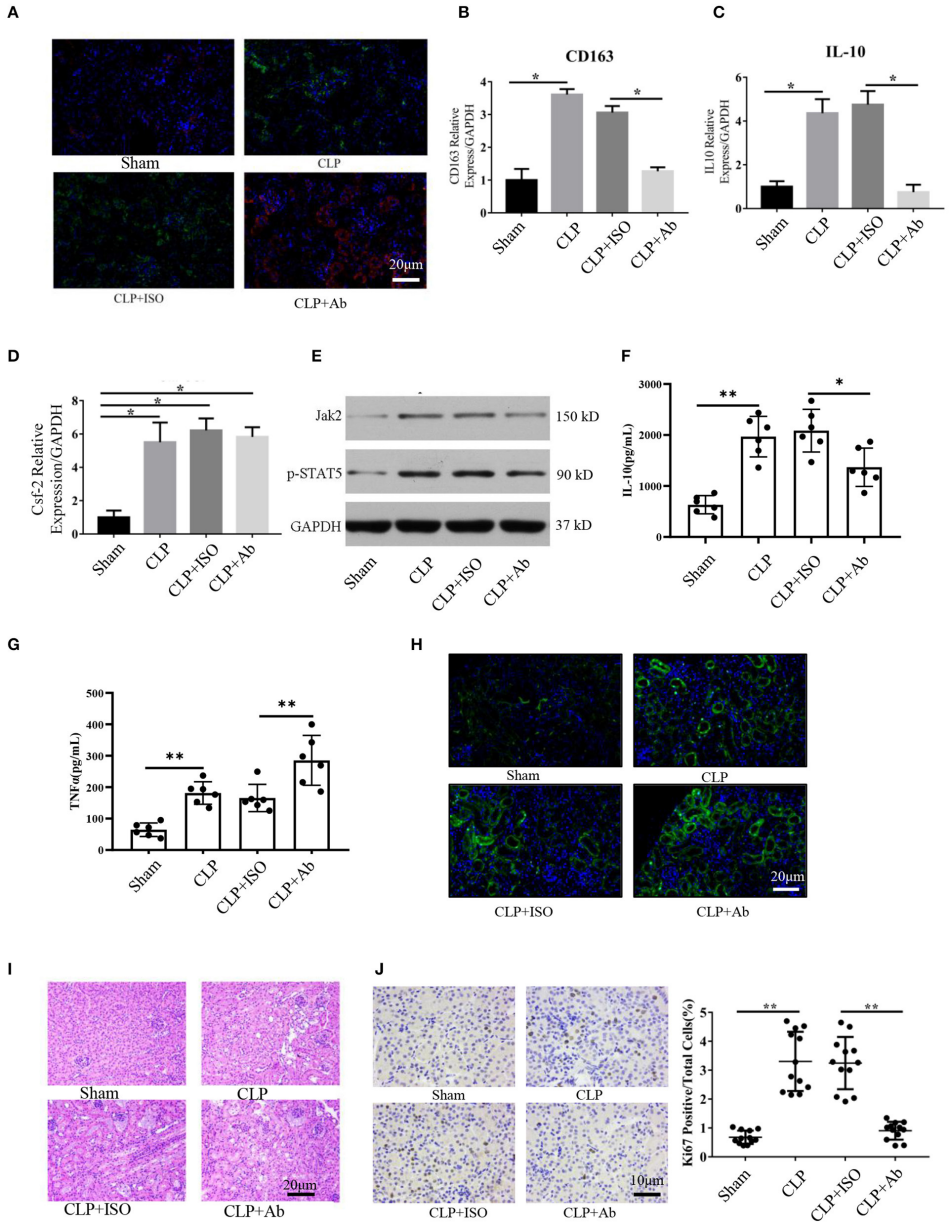
Blockade of Csf2 after CLP attenuated kidney macrophage alternative activation and slowed reparative tubule proliferation. **(A)** Serum and kidney tissues were collected for Scr and H&E staining at day 6. Representative immunofluorescence images of kidney tissue showing the expression of HLA-DR (M1 marker, red) and CD206 (M2 marker, green) after neutralizing Csf2 antibody or isotype antibody injection. Data are shown as the mean ± SD of 6 mice per group per experiment and were pooled from two independent experiments. **(B–D)** The indicated M2 markers (CD163 and IL10) and Csf2 were assessed using qRT-PCR. Data are shown as the mean ± SD of six mice per group per experiment, and two independent experiments were performed. *p < 0.05; two-tailed unpaired Student's *t*-test. **(E)** Western blotting was utilized to detect total STAT5 and phosphorylated STAT5 in the kidney cortex. Six mice were used per group per experiment, and two independent experiments were performed. **(F,G)** The concentrations of TNFα and IL10 in the kidney cortex were assayed by ELISA. Data are shown as the mean ± SD of 6 mice per group per experiment. **(H)** The apoptosis rate was analyzed in the kidneys of mice by TUNEL. Data are shown as the mean ± SD of 6 mice per group per experiment. **(I)** The collected kidneys from the four groups were stained with H&E. Scale bars: 20 μm. Kidney histology from CLP mice showing significant tubular epithelial swelling and brush border injury. Data are shown as the mean ± SD of six mice per group per experiment and were pooled from two independent experiments. **(J)** Photomicrograph of kidney tissue sections immunohistochemically stained for Ki67. Dense brown nuclear immunohistochemical staining indicates Ki67-positive status. Quantification of Ki67-positive tubular cells in the outer medulla. Data are shown as the mean ± SD of six mice per group per experiment and were pooled from two independent experiments, **p < 0.01; two-tailed unpaired Student's *t*-test.

Our data indicate that Csf2 could promote the transformation from M1 to M2 macrophages *in vitro*. Therefore, we hypothesized that blocking Csf2 signaling may inhibit macrophage transition in the kidneys of septic mice. To test whether Csf2 signaling also regulates macrophage transition *in vivo*, we first tested the expression of markers of M1 and M2 macrophages in kidney tissue. Double HLA-DR/CD206 immunofluorescence staining indicated that CD206 (red, M2 marker) expression was decreased after Csf2 antibody injection (Figure 5A), which indicated a decline in kidney macrophage transition toward the M2 phenotype after neutralizing antibody administration. In addition, the M2 markers CD163 and IL-10 were significantly reduced in the kidney cortex following Csf2 antibody treatment compared with isotype antibody treatment (Figures 5B,C), whereas the mRNA expression of Csf2 remained unchanged in these two groups (Figure 5D). Western blotting was used to detect the expression of p-STAT5 and Jak2. p-STAT5 and Jak2 activation decreased in the kidney cortex after anti-Csf2 antibody administration (Figure 5E).

To better and more objectively characterize the effect of Csf2 on M2, we detected the concentrations of IL-10 and TNFα in kidney tissue. As shown in Figures 5F,G, we found that the concentration of TNFα was much higher in the CLP + Ab group than in the CLP + ISO group, while the concentration of IL-10 was decreased following Csf2 antibody administration. The TUNEL assay was performed to detect the apoptosis rate. Kidneys from mice treated with the Csf2-neutralizing antibody showed a marked increase in the number of TUNEL-positive tubular epithelial cells (Figure 5H). We then attempted to confirm the histopathological damage among the different groups. H&E staining was performed on kidney tissue slices. H&E staining showed that tubular epithelial swelling and brush border injury were even worse in the CLP + Csf2 Ab group than in the CLP + ISO group (Figure 5I). AKI with proximal tubule death is usually followed by a wave of tubular proliferation, peaking at 48 h after injury, to restore tubular cell mass. The surviving epithelial cells have an equivalent capacity for repair (29, 30). Ki67, a marker of proliferation, was detected to characterize the repair process. The percentage of Ki67-positive cells was significantly decreased by Csf2–neutralizing antibody injection compared with isotype antibody injection after CLP (Figure 5J). These findings support our hypothesis that the Csf2 antibody significantly inhibited macrophage transition toward the M2 phenotype via the JAK2-STAT5 pathway and reduced tubular cell repair.

## Discussion

AKI is a global health problem with high morbidity and mortality (31). Macrophages are highly heterogeneous in terms of origin and function. Different subpopulations play various roles at different stages in the course of renal disease. Accumulating evidence has suggested that macrophages shift from an M1-like proinflammatory state during the early phase after injury to an M2-like reparative state during the tubular recovery phase. The signals that instruct macrophages to alter their gene expression profiles and promote tubular repair after septic AKI are unknown. Here, we revealed the molecular mechanism by which kidney tubular cells induce macrophage transformation. We found that Csf2 derived from tubular cells induced M0/M1-to-M2 differentiation and subsequently played a protective role in sepsis-induced AKI. Our findings suggest a novel therapeutic target for septic AKI.

Csf2 was one of the top 2 differentially expressed cytokines in the M1 + LPS-HK-2 group compared with the M1 group. Csf2 has been reported to regulate complement- and antibody-mediated phagocytosis, antigen presentation, leukocyte chemotaxis, microbicidal capacity, dendritic cell homeostasis, and adhesion (32, 33). Csf2 is produced by a variety of cells, such as fibroblasts, macrophages, endothelial cells, DCs, neutrophils, T cells, and tissue-resident cells, during inflammatory/autoimmune reactions. Additionally, Csf2 has various effects on the regulation of myeloid populations, including survival, activation, differentiation, and mobilization (34, 35). Csf2-dependent inflammatory pathways in monocytes (and macrophages) are likely to be critical for the purported role of Csf2 in inflammation, autoimmunity and host defense (36). However, the role of Csf2 in sepsis-induced AKI remains largely unknown. We found that administration of Csf2 in the coculture system decreased the apoptosis rate of HK-2 cells. Furthermore, fewer M1 macrophages migrated to the lower chamber, and the AKI biomarker TIMP-2 was also decreased in the culture medium. These results demonstrate a protective role of Csf2 in LPS-induced kidney injury. Basal levels of Csf2 are low under homeostatic conditions but can be quickly elevated during infection or inflammation. Modulating the functional homeostasis of macrophages can be of great benefit for sepsis-induced AKI (37). Therefore, it is reasonable to speculate that Csf2 could play a central role in mitigating sepsis-induced AKI.

Surprisingly, the source of Csf2 in our model system was exclusively secretion by HK-2 cells, not macrophages. Furthermore, Csf2 induced the M2 transition in a dose-dependent manner at doses up to 25 ng/ml, and 3 days were required for M1-M2 transition after exposure to Csf2 *in vitro*. In Fleetwood et al.'s study, Csf2-dependent murine bone marrow-derived macrophages (BMM) produced significantly more TNF-α and IL-6 compared with Csf1-dependent murine BMM. Based only on increased expression of pro-inflammatory cytokines, Csf2-treated monocytes/macrophages after exposure with LPS produce more cytokines have been termed “M1-like” (38). However, Csf2 is often used to generate DCs populations in BMM (39–41), and DCs express TLR2 and TLR4 (42, 43). No wonder that Csf2-dependent BMM secreted more proinflammatory cytokines after exposure to LPS. In our study, M1 macrophages polarization was induced with THP-1 cells by incubation with LPS and IFN-γ according to routine protocol (44, 45). After stimuli macrophage expressed the typical M1 makers, and Csf2 induced the transformation from M1 to M2. These results are consistent with the pathological process, in which proinflammatory macrophages (M1) predominate during the early injury phase (first 24–48 h), when tubular apoptosis is prominent, and local macrophages begin to express M2 markers during the tubular repair phase (3–5 days), when tubular epithelial cells are growing and remodeling the damaged nephron (46, 47).

Disturbances in macrophage plasticity might compromise immune responses and lead to the development of disease conditions (25). The phenotypic changes and functions of macrophages are regulated by various signaling pathways. Transcription factors, including MAPK, JNK, and NF-κB, are involved in M1 programming (48). PPARγ-deficient macrophages are resistant to M2 polarization and promote insulin resistance (49). IRF, C/EBPβ and STAT regulate the M2 phenotype (26). The core factors that control macrophage phenotype and function in sepsis-induced AKI are still unclear. Our results showed that the Jak2-STAT5 pathway was involved in tubular cell-mediated macrophage transition. The Csf2 receptor, a dodecamer structure, was activated after Csf2 treatment, recruited Jak2 and led to downstream activation of STAT5, thus promoting the M1-to-M2 transition. The Jak2-STAT5 pathway was activated at 24 h and reached a peak at 48 h after Csf2 treatment. We identified tubule-derived Csf2 as a STAT5 activator mediating the macrophage transition from a proinflammatory to a proreparative phenotype.

Because Csf2 promotes the transition from the M1 phenotype to the M2 phenotype *in vitro*, we also examined whether a Csf2 antibody or recombinant Csf2 could contribute to macrophage differentiation in a murine sepsis model. Csf2 blockade through antibodies strongly inhibited M1 macrophage differentiation and increased mortality in CLP-induced septic kidney injury. Consistent with the data obtained in mice subjected to injection of a Csf2-neutralizing antibody, Csf2 protein administration improved the survival rate, suggesting that Csf2 signaling may promote the transition to M2 macrophages. M2 macrophages attenuate sepsis-induced AKI by upregulating IL-10 expression and suppressing TNF-α secretion. Moreover, the reparative process in kidney injury was inhibited following administration of the Csf2 antibody. Our result is also consistent with a randomized controlled trial of 39 patients with sepsis who had immune dysfunctions: Csf2 therapy in these patients was safe and effectively restored the immunocompetence of monocytes (50).

We have sufficient supporting evidence to propose that tubule cell-secreted Csf2 induces macrophage transformation and protects against AKI, although there are some limitations to our study. Previous research has reported that the production of Csf2 is induced by proinflammatory cytokines such as IL-1α, IL-1β, IL-12, and TNF and inhibited by IL-4, IFN-γ, and IL-10 (51, 52). In our study, Csf2 was increased at an early time point (18 h) in the kidney, but the stimulus resulting in Csf2 overexpression remains unknown. As Csf2 may serve as a potential therapeutic target for sepsis-induced AKI, host defense parameters, including circulating cytokines and the bacterial load, should be evaluated in a CLP model following Csf2 treatment. Moreover, whether other organs are affected by Csf2 treatment is unknown and must be further investigated.

## Conclusions

**Figure 6:**
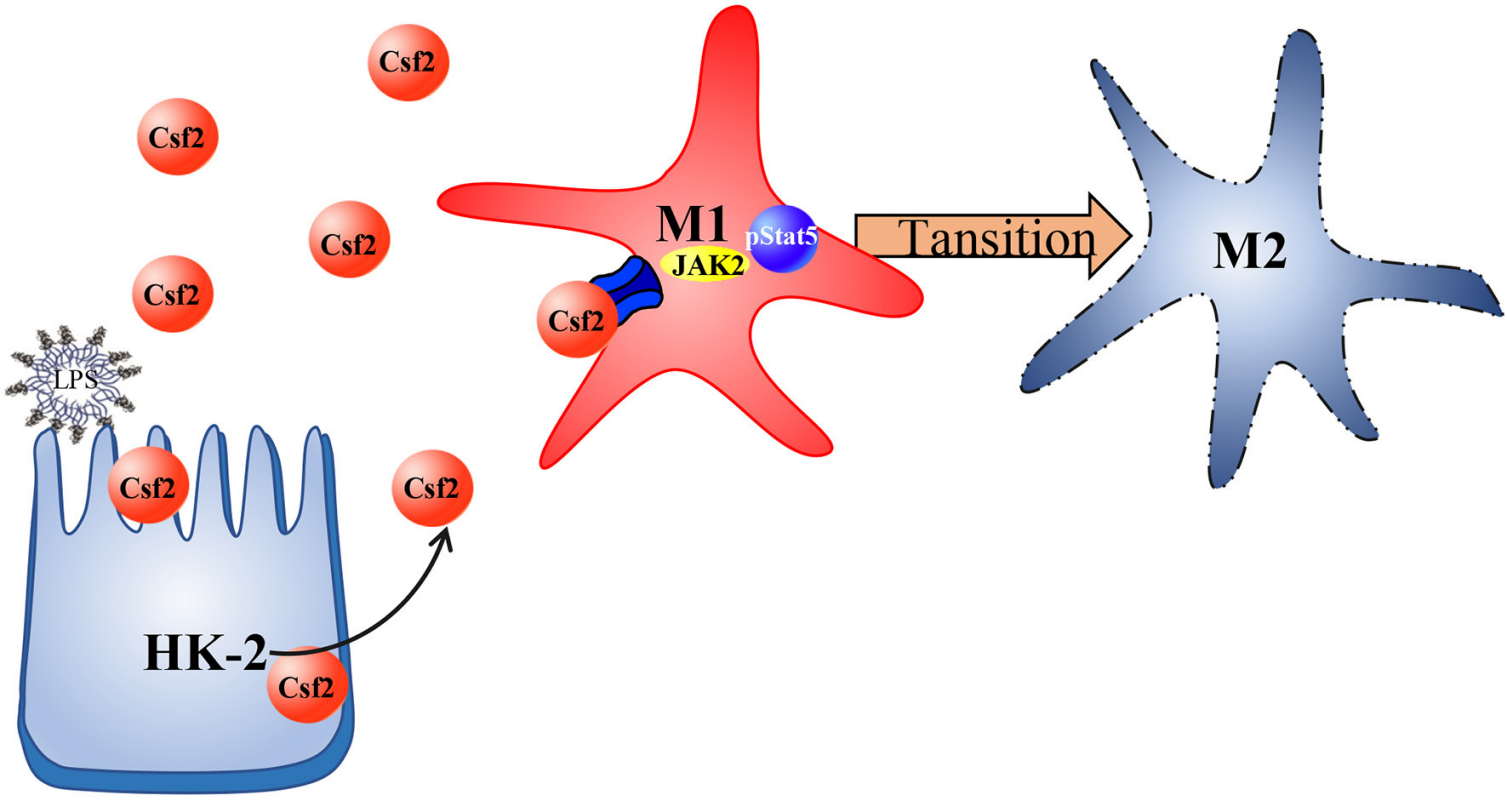
Schematic representation of Csf2 regulation of macrophage transformation in sepsis-induced AKI. Csf2 secreted by tubular cells promotes Stat5 phosphorylation, reduces cell apoptosis, facilitates injured tubular cell repair and therefore attenuates kidney injury *in vitro* and *in vivo*.

In summary, we investigated the regulatory function of Csf2 in sepsis-induced AKI and found that Csf2 secreted by HK-2 cells could promote macrophage transition toward the M2 phenotype via the p-STAT5 signaling pathway. Activated proinflammatory macrophages became polarized to the M2 phenotype, which dampens the proinflammatory response. Csf2 treatment reduced tubular cell apoptosis and subsequently improved AKI and survival (Figure 6). Csf2-mediated macrophage transition is an important mechanism of renal epithelial cell repair after kidney injury and could provide an alternative method for the treatment of septic AKI in the future.

## Data Availability Statement

All datasets generated for this study are included in the article/Supplementary Material.

## Ethics Statement

The animal study was reviewed and approved by Animal Care and Use Committee of Wuhan University.

## Author Contributions

YL: conceived the project, designed the project, extract and analyzed data, drafted the manuscript, and approved the final manuscript. YZ: drafted the part of the discussion and background of the manuscript. YL, PZ, and JZ: conducted the experiments. JK and ZP: designed the project, edited the manuscript, and approved the final version. All authors contributed to the article and approved the submitted version.

## Conflict of Interest

JK discloses grant support and consulting fees from Astute Medical. The remaining authors declare that the research was conducted in the absence of any commercial or financial relationships that could be construed as a potential conflict of interest.

## Supplementary Material

Supplementary Table 1 The absolute value of cytokines and chemokines in the supernatant.
